# Molecular recognition of a membrane-anchored HIV-1 pan-neutralizing epitope

**DOI:** 10.1038/s42003-022-04219-6

**Published:** 2022-11-18

**Authors:** Johana Torralba, Igor de la Arada, Angélica Partida-Hanon, Edurne Rujas, Madalen Arribas, Sara Insausti, Claire Valotteau, Javier Valle, David Andreu, José M. M. Caaveiro, María Angeles Jiménez, Beatriz Apellániz, Lorena Redondo-Morata, José L. Nieva

**Affiliations:** 1 Instituto Biofisika (CSIC-UPV/EHU), University of the Basque Country (UPV/EHU), PO Box 644, 48080 Bilbao, Spain; 2 Department of Biochemistry and Molecular Biology, University of the Basque Country (UPV/EHU), PO Box 644, 48080 Bilbao, Spain; 3 Institute of Physical Chemistry “Rocasolano” (IQFR-CSIC), Serrano 119, E-28006 Madrid, Spain; 4 Laboratoire Adhesion et Inflammation, INSERM U1067, CNRS UMR 7333, Aix-Marseille Université, Turing Centre for Living systems, Marseille, France; 5 Laboratory of Proteomics and Protein Chemistry, Department of Medicine and Life Sciences, Pompeu Fabra University, Barcelona Biomedical Research Park, Dr. Aiguader 88, 08003 Barcelona, Spain; 6 Laboratory of Global Healthcare, School of Pharmaceutical Sciences, Kyushu University, Fukuoka, 819-0395 Japan; 7 Department of Physiology, Faculty of Pharmacy, University of the Basque Country (UPV/EHU), Paseo de la Universidad, 7, 01006 Vitoria-Gasteiz, Spain; 8 Université de Lille, CNRS, Inserm, CHU Lille, Institut Pasteur de Lille, U1019-UMR9017/CIIL-Centre d′Infection et d′Immunité de Lille, F-59000 Lille, France; 9 Present Address: Ikerbasque, Basque Foundation for Science, 48013 Bilbao, Spain; 10 Present Address: Pharmacokinetic, Nanotechnology and Gene Therapy Group, Faculty of Pharmacy, University of the Basque Country UPV/EHU, 01006 Vitoria-Gasteiz, Spain; 11 Present Address: Bioaraba, Microbiology, Infectious Disease, Antimicrobial Agents, and Gene Therapy, 01006 Vitoria-Gasteiz, Spain

**Keywords:** Nanoscale biophysics, Membrane biophysics, Immunology

## Abstract

Antibodies against the carboxy-terminal section of the membrane-proximal external region (C-MPER) of the HIV-1 envelope glycoprotein (Env) are considered as nearly pan-neutralizing. Development of vaccines capable of producing analogous broadly neutralizing antibodies requires deep understanding of the mechanism that underlies C-MPER recognition in membranes. Here, we use the archetypic 10E8 antibody and a variety of biophysical techniques including single-molecule approaches to study the molecular recognition of C-MPER in membrane mimetics. In contrast to the assumption that an interfacial MPER helix embodies the entire C-MPER epitope recognized by 10E8, our data indicate that transmembrane domain (TMD) residues contribute to binding affinity and specificity. Moreover, anchoring to membrane the helical C-MPER epitope through the TMD augments antibody binding affinity and relieves the effects exerted by the interfacial MPER helix on the mechanical stability of the lipid bilayer. These observations support that addition of TMD residues may result in more efficient and stable anti-MPER vaccines.

## Abstract (web summary)

The molecular recognition of the carboxy-terminal section of the membrane-proximal external region (C-MPER) of the HIV-1 envelope glycoprotein in membrane mimetics is characterised to help shape continued development of more efficient and stable anti-MPER vaccines.

## Introduction

The Env glycoprotein sequence juxtaposed to the external leaflet of the viral membrane, known as the Membrane-Proximal External Region (MPER, Env residues 656–683; HXB2 numbering), is the target for a class of HIV-1 broadly neutralizing antibodies (bnAbs)^1–5^. In particular, those that target the highly conserved C-terminal section (C-MPER, Env residues 671–683; HXB2 numbering), which precedes the transmembrane domain (TMD), steadfastly exert the broadest levels of neutralization among identified HIV-1 bnAbs, and are thus considered as nearly pan-neutralizing^6–12^. Production through vaccination of antibodies with comparable neutralization breadth would potentially confer full protection against infection by the tremendous diversity of circulating HIV-1 variants, a consideration that justifies the interest in elucidating the mechanisms that underlie the generation and antiviral activity of C-MPER-targeting bnAbs^1–3,5^.

The immune system is capable of eliciting bnAbs with C-MPER specificity during infection and, so far, isolated bnAbs targeting this site exhibit comparable modes of C-MPER helix recognition, two facts that taken together support the existence of at least one stable MPER ensemble recognized by B-cell receptors^5,8,10,13^. However, the composition and structure of that hypothetical ensemble is still a matter of debate. The abundance, periodicity and conservation degree of aromatic residues within the sequence can theoretically stabilize a mainly helical conformation of MPER on membrane-interface contact^14–20^. Based on this arrangement, a model assumes that anti-MPER bnAbs would dock onto a partially buried MPER helix adsorbed in parallel to the membrane plane, and extract it from its interfacial position^20–24^.

A different view derives from the identification of a chain kink located at the upstream Env position 671–673, which establishes two helical subregions within MPER, i.e., N-MPER and C-MPER^8,11,25–31^. Structure resolution through X-ray diffraction of different Fabs with bound lipids and epitope-peptides^10,11,32,33^, in combination with cryo-electron microscopy (cryo-EM) reconstructions of Env-Fab complexes^10,27,34^, suggests that C-MPER would likely compose the N-terminus of the continuous TMD helix and stick out with its main axis almost perpendicular to the membrane plane in a ‘pole’-like fashion. In this arrangement, the C-MPER epitope would become accessible for recognition by the Fabs, which would engage laterally with the helix pole, making extensive contacts with the membrane through an accommodating surface^10,11,29,33,34^.

Here, using a combination of structural and biophysical techniques, we assess the role of conserved TMD helix residues in the C-MPER epitope recognition process that evolves in a membrane environment. We selected the antibody 10E8 as a model for these studies, as this is the most potent and best characterized C-MPER-targeting bnAb^8,27,29,33–38^. We presented the 10E8 epitope in detergent micelles and lipid bilayers in two formats: (i) as the C-terminal section of an interfacial helix (N/C-MPER peptides), i.e., emulating constructs that are profusely utilized as components of experimental MPER vaccines^2,17,39^; and (ii) as the N-terminus of the Env TMD helix (C-MPER-TMD peptides). Our comparative evaluation supports that addition of TMD residues to peptide-based vaccine formulations would be required for the production 10E8-like MPER bnAbs.

## Results

### Rationale for the selection of peptide sequences and membrane systems

**Fig. 1:**
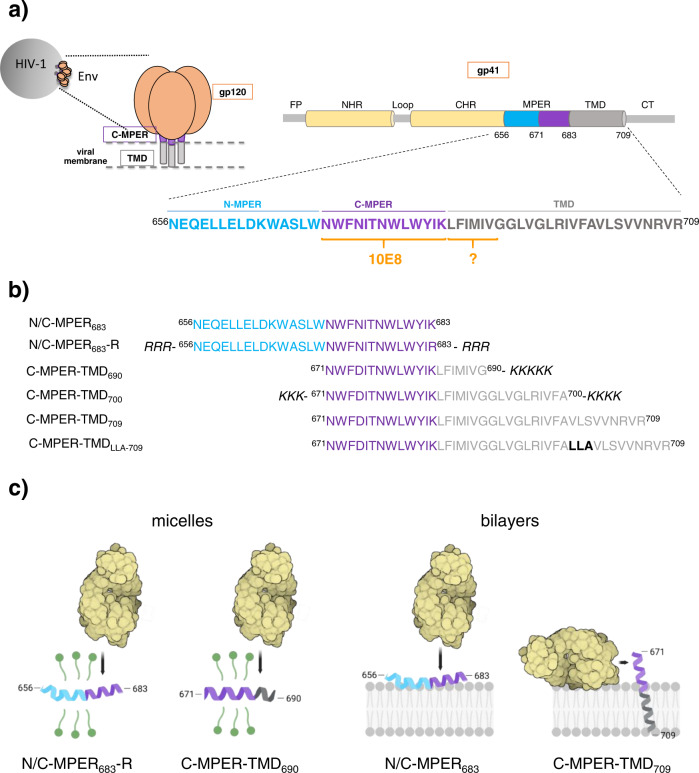
Peptides and model systems selected to study 10E8 binding to the C-MPER helix in membrane mimetics. **a** Sequence and range of N-MPER, C-MPER and TMD domains within the HIV-1 Env subunit gp41. The orange line denotes the sequence spanning the nominal 10E8 epitope. The question mark raises the possible contribution of TMD residues. The diagram on top depicts the different domains of gp41: FP, fusion peptide; NHR and CHR, amino- and carboxy-terminal helical regions, respectively; MPER, membrane-proximal external region; TMD, transmembrane domain; CT, cytoplasmic domain. **b** Sequence and designation of the N/C-MPER and C-MPER-TMD-based peptides used in this study. **c** Models for recognition of the C-MPER helix by the Fab 10E8 in detergent micelles or inserted into phospholipid bilayers. The latter are depicted as interfacial or membrane-spanning helices. (Created with BioRender.com).

Figure 1a depicts the position and sequence range of the HIV-1 gp41 MPER-TMD region. To assess the possible contribution of TMD residues to C-MPER helix recognition in membrane environments, we measured 10E8 binding to a series of C-MPER-containing peptides (Fig. 1b), both, in the presence of detergent micelles, or after reconstitution in lipid bilayers (Fig. 1c).

**Fig. 2:**
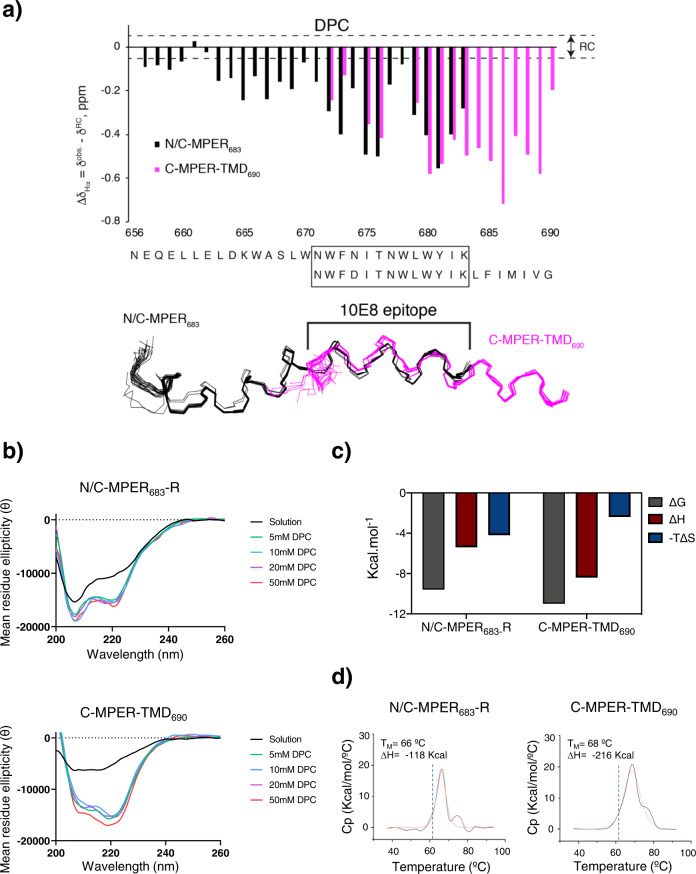
Binding energetics of Fab 10E8 to helical C-MPER epitope in presence of DPC micelles. **a** Comparison of NMR structures of N/C-MPER_683_^19^ and C-MPER-TMD_690_ in presence of DPC micelles. Top: Hα conformational shifts (Δδ_Hα_, ppm) observed for both peptides in 20 mM DPC micelles, pH 7.0. Dashed lines indicate the random coil (RC) range. Bottom: superposition of the 3D structural models supporting the adoption of similar conformations by the 10E8 epitope sequence in both peptides. **b** CD spectra obtained for N/C-MPER_683_−R and C-MPER-TMD_690_ at 25 °C in the presence of increasing concentrations of DPC. **c** Thermodynamic signatures of the binding of Fab 10E8 and peptides N/C-MPER_683_-R or C-MPER-TMD_690_ determined by ITC in the presence of 5 mM DPC. **d** Stability of Fab with peptide bound. DSC scans of 10E8 in the presence of N/C-MPER_683_-R or C-MPER-TMD_690_ were collected between 35 and 95 ºC. The values  of the melting temperature (*T*_M_) of the main transition and unfolding enthalpy (Δ*H*) are shown. Black and red lines represent raw data and fitting curves, respectively. The dotted vertical blue lines indicate the *T*_M_ value of the apo form of 10E8. Thermodynamic parameters shown for peptide C-MPER-TMD_690_ (**c**, **d**) as previously reported in our previous study^29^. These values were obtained under the same experimental conditions as the ones used in the current study.

In the presence of detergent, we compared binding features of two types of peptide surrogate (Fig. 1c, left panel). On the one hand, we used the peptide N/C-MPER_683_, which includes MPER N- and C-terminal subregions^19^, and N/C-MPER_683_-R, a derivative containing a solubility 6 R tag, which was similar in sequence to the epitope peptide used for the initial structural and binding affinity characterizations of the antibody 10E8^8^. Notably, N/C-MPER_683_-like peptides are currently used for immunogen design^17,19,40,41^, and as molecular baits for detection of MPER-positive B cells in infected individuals and vaccinated animals^9,10,42,43^. On the other hand, we used the elongated peptide C-MPER-TMD_690_ to emulate the continuous helix solved in complex with the Fab 10E8 by X-ray diffraction^29^_._ This peptide incorporated N-terminal residues of the TMD and could be stabilized in the presence of dodecylphosphocholine (DPC) as a soluble C-MPER-TMD ligand (see Fig. 2b below).

Data derived from experiments in detergent are relevant for development of peptide-based micellar formulations as MPER-targeting vaccines^17,39^. However, cumulative evidence supports the hypothesis that, to attain broad neutralization, C-MPER-targeting bnAbs must develop the capacity to engage with the MPER helix protruding from the membrane surface (pole-like topology)^5,29,33,34,44^. In this context, a vaccine that accounts for the steric factors that condition recognition at the membrane surface is likely required for selecting the proper B-cell receptors from the repertoire targeting C-MPER.

Thus, we sought to reconstitute the C-MPER helix in lipid bilayers, and test the capacity of the Fab 10E8 to engage with its epitope under these conditions (Fig. 1c, right panel). In these experiments, we primarily compared the peptides N/C-MPER_683_ and C-MPER-TMD_709_ (Env residues 671–709, HXB2 numbering), which included the nominal Env TMD sequence (Env residues 684–709)^45^. As shown in Fig. 1c (right), we expected these peptides to expose the 10E8 epitope with different topologies after their reconstitution in membranes, the former at the C-terminus of an interfacial helix, the latter as the N-terminus of a continuous helix that spans the membrane.

In addition, two peptides that combined the 10E8 epitope with shorter or longer TMD moieties were used to probe the topology adopted in membranes upon reconstitution, namely: C-MPER-TMD_700_ (Env residues 671–700, HXB2 numbering), containing a minimal TMD anchor as described by Hunter and cols^46^, and Lys-tags added at both ends of the molecule to increase its solubility^26,47^; and C-MPER-TMD_LLA-709_, a sequence elongated by adding an additional helical turn to the section spanning the hydrophobic membrane core (sequences displayed in Fig. 1b).

### 10E8 binding to MPER and C-MPER-TMD helix surrogates in the presence of detergent micelles

To prove that in the C-MPER-TMD_690_ peptide the 10E8 epitope adopted a helical conformation comparable to that previously described to occur in N/C-MPER_683_ by our group^19^, we first carried out its structural characterization by solution NMR spectroscopy in non-polar media provided by 1,1,1,3,3,3-hexafluoro-2-propanol (HFIP) and DPC micelles (Fig. 2a, Table 1 and Supplementary Figs. 1, 2). Figure 2a compares the sequences, Hα conformational shifts and superposition of the NMR structures calculated for C-MPER-TMD_690_ in the presence of DPC micelles with those previously calculated for N/C-MPER_683_^19^. The similitudes of Hα conformational shifts and structural ensembles, confirmed that in both peptides the sequence spanning the nominal 10E8 epitope adopted an equivalent helical conformation (see also Supplementary Fig. 2 for the comparison in HFIP).

**Table 1:** NMR and refinement statistics for structure of C-MPER-TMD_690_ in DPC (20 mM deuterated dodecylphosphocholine, 2 mM HEPES pH 7.0, H_2_O/D_2_O 9:1 v/v) and HFIP (25% deuterated 1,1,1,3,3,3-hexafluoro-2-propanol in 2 mM HEPES pH 7.0, H_2_O/D_2_O 9:1 v/v).

	C-MPER-TMD_690_ in DPC (8B6X)^a^	C-MPER-TMD_690_ in HFIP (8B6Y)^a^
**NMR distance and dihedral constraints**		
Distance constraints		
Total NOE	375	356
Intra-residue	175	163
Inter-residue	200	193
Sequential (|*i* − *j*| = 1)	75	96
Medium-range (|*i* − *j*| ≤ 5)	125	97
Long-range (|*i* – *j* | > 5)	0	0
Intermolecular hydrogen bonds	0	0
Total dihedral angle restraints	32	45
ϕ	17	23
ψ	15	22
**Structure statistics**		
Violations (mean and s.d.)		
Distance constraints (Å)	0.03 ± 0.00	0.02 ± 0.00
Dihedral angle constraints (°)	0.4 ± 0.1	0.04 ± 0.00
Max. dihedral angle violation (°)	0.6	0.04
Max. distance constraint violation (Å)	0.4	0.02
Deviations from idealized geometry		
Bond lengths (Å)	0	0
Bond angles (°)	0	0
Impropers (°)	0	0
Average pairwise r.m.s. deviation^b^ (Å)		
Heavy	1.9 ± 0.6 (0.7 ± 0.4)^c^	0.7 ± 0.2 (0.3 ± 0.1)^c^
Backbone	2.9 ± 0.7 (1.4 ± 0.6)^c^	1.7 ± 0.3 (1.3 ± 0.3)^c^

^a^PDB codes are given in parenthesis.

^b^Pairwise r.m.s. deviations were calculated among 20 structures.

^c^Values in parenthesis are for residues 672–690.

However, the NMR signals of C-MPER-TMD_690_ obtained in DPC solution were broader than those obtained for N/C-MPER_683_. This observation would be consistent with the existence of a monomer/oligomer equilibrium, more probable in the case of the C-MPER-TMD_690_. The CD spectra obtained in samples containing increasing DPC concentrations would support that possibility (Fig. 2b). In both samples, a DPC concentration of 5 mM was sufficient to promote a soluble helical conformation of the peptides. However, the 222/208 absorbance ratios were clearly higher in the spectra of C-MPER-TMD_690_, following a pattern observed in coiled coils and helical oligomeric structures^48–50^.

Having confirmed that 5 mM DPC is enough to solubilize the peptides, we next determined the affinity of the antibody by isothermal titration calorimetry (ITC) in the presence of the detergent. The titration of C-MPER-TMD_690_ and N/C-MPER_683_-R showed a 1:1 binding stoichiometry to the antibody (Supplementary Fig. 3). However, the binding affinity of antibody 10E8 to C-MPER-TMD_690_ (evaluated from the dissociation constant (*K*_D_)) was nearly an order of magnitude higher than that to N/C-MPER_683_-R (*K*_D_ = 10 ± 1.6 nM and 93 ± 13 nM, respectively) (Fig. 2c and Supplementary Fig. 3). Moreover, the binding signature of C-MPER-TMD_690_ indicated a significantly more favorable binding enthalpy (Δ*H* = −8.4 ± 0.1 kcal mol^−1^) than that for N/C-MPER_683_-R (Δ*H* = −5.4 ± 0.1 kcal mol^−1^), suggesting stronger and more durable interactions of the antibody with the peptide derived from the C-terminal subregion of MPER. Predictably, the entropic component of the interaction contributed less to binding in C-MPER-TMD_690_ (−*T*Δ*S* = −2.4 ± 0.2 kcal mol^−1^) than in N/C-MPER_683_-R (−4.2 ± 0.2 kcal mol^−1^).

Similarly, differential scanning calorimetry (DSC) revealed that the melting temperature (*T*_*M*_) of the Fab 10E8^29^ (*T*_M_ of 61.5 °C) was higher upon complex formation with C-MPER-TMD_690_ (*T*_*M*_ = 68 ± 1.0 °C) that with N/C-MPER_683_-R (*T*_M_ = 66 ± 1.0 °C) (Fig. 2d). Higher stabilization upon C-MPER-TMD_690_ binding was also observed from the increase unfolding enthalpy (Δ*H*_DSC_) determined with this peptide. While binding to C-MPER-TMD_690_ induced a Δ*H*_DSC_ = −216 kcal mol^−1^, the unfolding enthalpy of the 10E8 Fab-N/C-MPER_683_-R complex was 96 kcal lower (Δ*H*_*DSC*_ = −118 kcal mol^−1^). Overall, the energetics that govern Fab 10E8 binding support the benefit of including residues from the N-region of the TMD for a higher affinity and specificity toward its epitope.

### Recognition of C-MPER helices reconstituted in lipid vesicles

**Fig. 3:**
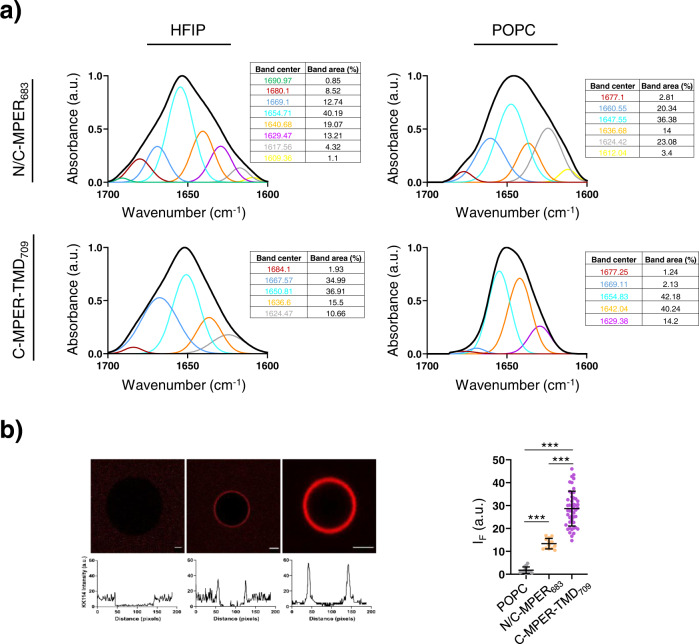
10E8 recognition of C-MPER helices reconstituted in POPC vesicles. **a** Reconstitution of the peptides in lipid bilayers. IR absorption bands in the amide I region obtained for peptides in 50% HFIP (v:v) (left panels) or after reconstitution in POPC membranes. Percentages contributed by each band component are displayed in the side Tables. The peptide-to-lipid ratio was 1:50 (mol:mol). **b** Binding of Fab 10E8 to single POPC vesicles containing the peptides reconstituted. Left: Confocal microscopy images of single POPC vesicles with or without the peptides and incubated with KK114-Fab 10E8 (red label). Traces below follow the changes in KK114 fluorescence intensity at the equatorial plane. Right: KK114-Fab 10E8 intensities measured in the different GUV samples. In samples containing peptide the peptide-to-lipid ratio was 1:250 (mol:mol). Scale bars are 2 µm. Number of vesicles *n* ≥ 13. Center line, mean; whiskers SD (Mann–Whitney Test: ***p < 0.001; **p < 0.01, *p < 0.05, n.s. ≥0.05).

For the reconstitution in 1-palmitoyl-2-oleoylphosphatidylcholine (POPC) membranes, the peptides N/C-MPER_683_ and C-MPER-TMD_709_ dissolved in an HFIP solution were mixed with lipids in organic solvent and desiccated. The resulting peptide-lipid films were subsequently subjected to gentle hydration^47^. The secondary structure determination by Infrared (IR) spectroscopy confirmed absorption maxima around 1650 cm^−1^ in the amide-I region of the spectra, consistent with the efficient reconstitution of both peptides adopting main α-helical conformations in POPC bilayers (Fig. 3a). However, as judged from the width of the bands, when compared with the spectra obtained in 50% HFIP, a more pronounced reduction of conformational flexibility (i.e., lower number of absorption modes) could be discerned in the case of the reconstituted C-MPER-TMD_709_ peptide. Moreover, a band component centered at 1642 cm^−1^ markedly contributed to the absorption by this peptide. Coiled coils exhibit amide-I band components centered at ca. 1640 and 1630 cm^−1^ ^51^. Thus, the presence and intensity of similar components in the amide-I spectra of C-MPER-TMD_709_ would be compatible with oligomer formation upon reconstitution of this peptide in membranes.

Besides the adoption of main helical structures, vesicle-flotation experiments corroborated the total incorporation of both peptides into membranes following the reconstitution procedure (Supplementary Fig. 4). Thus, we next compared the accessibility to the 10E8 epitope in N/C-MPER_683_ and C-MPER-TMD_709_ α-helices reconstituted in membranes at comparable densities (Fig. 3b). To that end, we quantified antibody binding to single vesicles by confocal microscopy of Giant Unilamellar Vesicles (GUVs) incubated with fluorescently labeled Fab 10E8^36,47^. According to the scored intensities, the Fab 10E8 appeared to bind more efficiently to C-MPER-TMD_709_-containing vesicles than to the N/C-MPER_683_-containing ones. Consistent with an epitope recognition-dependent phenomenon, upon incubation with the Fab, the KK114 probe was not detected in association with membranes of GUVs devoid of peptide.

### Topology of C-MPER helices reconstituted in lipid bilayers

**Fig. 4:**
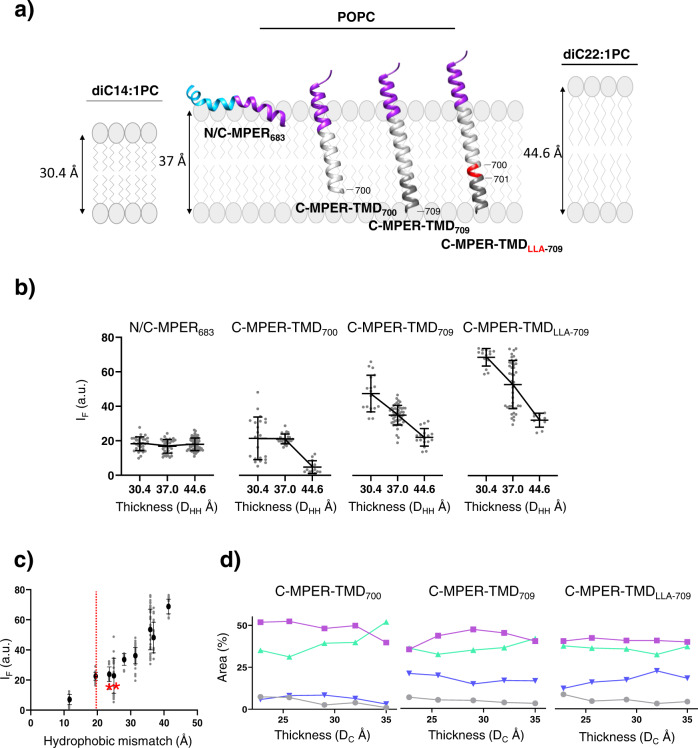
10E8 epitope accessibility in C-MPER helices reconstituted in lipid bilayers of different thicknesses. **a** Models for 10E8 epitope exposure upon increasing the length of the membrane-spanning scaffold or the bilayer thickness. The C-MPER and the TMD are colored in purple and gray respectively. The extra turn in C-MPER-TMD_LLA-709_ is depicted in red. **b** Binding of Fab 10E8 to C-MPER helices as a function of the headgroup-to-headgroup distance (D_HH_) parameter. **c** Binding extents as a function of the hydrophobic mismatch calculated as a function of the helix length and hydrocarbon core thickness (D_C_). The red line starts at 19.5 Å, i.e., the distance covered by the helical C-MPER epitope. **d** Effect of the lipid bilayer thickness on the conformation adopted by C-MPER-TMD helices reconstituted in lipid bilayers. The evolution of the different structural components (percentage of IR amide-I band area, see Fig. S5) are displayed as a function of the D_C_ parameter estimated by Gallová et al.^55^ for the series of monounsaturated PCs. Symbols and lines colored as follows: Purple: 1655 cm^−1^ (α-helix); Green: 1642 cm^−1^ (helix-helix interactions/disordered); Blue: 1630 cm^−1^ (solvated helices); Gray: 1672 cm^−1^ (turns).

It has been previously reported that certain MPER-TMD constructs may remain associated to the membrane interface, without traversing the lipid bilayer^24^. To ensure that following our reconstitution procedure the C-MPER helix adopted the assumed topologies in the lipid bilayer (see previous Fig. 1c), we performed antibody-binding assays as a function of the TMD length and membrane thickness (Fig. 4). Models displayed in Fig. 4a illustrate the different synthetic peptides derived from the HIV-1 C-MPER-TMD sequence that were employed in this approach and the rationale behind their application (see also Fig. 1).

Thus, to infer C-MPER helix topology, N/C-MPER_683_, C-MPER-TMD_700_, C-MPER-TMD_709_ and C-MPER-TMD_LLA-709_ were reconstituted in lipid bilayers made of diacyl PCs containing monounsaturated acyl chains of different lengths (see also Supplementary Figs. 5, 6). Based on the ‘pole’-like model proposed for 10E8 docking to Env in the pre-fusion state^27,29,33^, we reasoned that the length of the membrane-spanning scaffold and the thickness of the lipid bilayer should modulate epitope accessibility and, hence, affinity of the antibody, in the case of the transmembrane helices, but not in the case of helices adsorbed to the interface (Fig. 4a).

Thus, to establish the influence of the bilayer thickness on the accessibility to the 10E8 epitope, we selected lipid bilayers made of diC14:1PC and diC22:1PC, which are respectively thinner and thicker than those made of POPC (C16:0-C18:1PC)^52,53^. The experimental results on KK114-Fab binding to single vesicles revealed effects of bilayer thickness and TMD length for the peptides based on the C-MPER-TMD sequence (Supplementary Fig. 5), but these effects were not observed in the case of the peptide derived from the N/C-MPER sequence. The binding dependency on the actual head group-to-head group bilayer distance (D_HH_)^53,54^ is shown for all peptides in the Fig. 4b. Fab 10E8 binding to GUVs containing the C-MPER-TMD peptides followed two different trends. On the one hand, at a defined thickness, binding augmented upon increasing the TMD length of the peptide (i.e., C-MPER-TMD_LLA-709_ > C-MPER-TMD_709_ > C-MPER-TMD_700_). On the other hand, for a defined peptide, binding improved for the thinner membranes in comparison with the thicker ones (i.e., diC14:1PC > C16:0-C18:1PC > diC22:1PC). In sharp contrast, in the case of the reconstituted peptide N/C-MPER_683_ the measured binding extents were the same in the three types of vesicles, hence, independent of the distance D_HH_.

Another observation supporting an epitope-exposure phenomenon governed by the topology adopted by the TMD in membranes, is the nearly linear dependence of binding on the hydrophobic mismatch calculated with respect to bilayer hydrocarbon thickness D_C_^55^, which is observed in the case of the C-MPER-TMD-derived peptides (Fig. 4c).

In addition, the lipid bilayer distance covered by the hydrocarbon acyl chains strongly conditions the favorable interactions between TMDs and lipids, so that a mismatch between this distance and the TMD length can give rise to alterations in the structure and function of the integral membrane proteins^56^. Therefore, to rule out D_C_ effects on the conformations adopted by the reconstituted MPER-TMD peptides, we performed IR experiments and varied systematically this parameter utilizing a complete series of monounsaturated PCs (i.e., diC14:1PC, diC16:1PC, diC18:1PC, diC20:1PC and diC22:1PC)^55^. Figure 4d displays the changes in peptide conformation (area percentages covered by the amide I band components, see Supplementary Fig. S6) as a function of the hydrocarbon section thickness. We did not observe substantial changes in the conformations adopted by any of the peptides reconstituted in the systematically thicker lipid bilayers, supporting that the measured differences in antibody binding reflected the accessibility degree of the C-MPER helix at the membrane surface.

In conclusion, the differential effects of TMD length and bilayer thickness on Fab 10E8 binding are consistent with the C-MPER helix adopting different topologies after reconstitution of the N/C-MPER_683_ and C-MPER-TMD_709_ peptides in lipid bilayers; as a helix in contact with the membrane-interface in the former case, as the N-terminal end of a continuous TMD helix in the latter.

### Binding to C-MPER helices reconstituted in supported phospholipid bilayers assessed by Single-Molecule Force Spectroscopy

To measure quantitatively 10E8 affinity towards the C-MPER helices adopting alternative topologies in POPC bilayers, we turned to Atomic Force Microscopy (AFM) in its force spectroscopy (FS) format^57–59^. Specifically, we used single-molecule FS, an approach widely applied to characterize unbinding processes of antibody-antigen interactions^60–65^. Overall, this approach requires the measurement of the rupture forces of an antibody-antigen interaction at different loading rates, which allows to reconstruct the energy landscape of the interaction and the extrapolation to the dissociation rate at zero force representing the spontaneous *k*_off_ of the binding reaction at a given temperature^66,67^. Furthermore, the analysis provides information on the potential barrier width (*χ*_β_), a useful parameter for scoring conformational changes in the binding site or other rearrangements of the interacting molecules when comparing different unbinding partners.

**Fig. 5:**
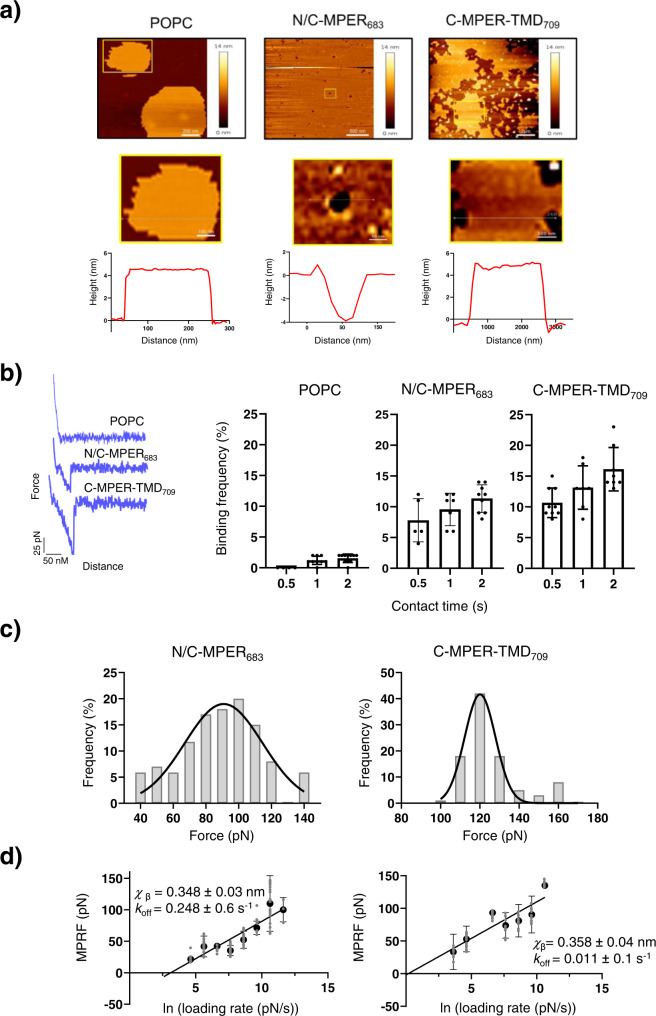
Single-molecule force spectroscopy assays to evaluate Fab 10E8 binding strength. **a** Topographical analysis by AFM of POPC SPBs containing C-MPER helices reconstituted. AFM images were taken from POPC SPBs with or without peptide as indicated in the panels. Plots below images display the height profiles across the trajectories indicated by the white lines. **b** Unbinding events scored as a function of the contact time. The plot on the left compares selected examples of experimental Force-distance curves displaying single events in POPC:N/C-MPER_683_ and POPC:C-MPER-TMD_709_ samples, but not in control POPC samples devoid of peptide. Plots on the right compare the binding frequencies after probing different SPBs at various contact times. The retraction velocity was fixed in these experiments (2 μm s^−1^). **c** Comparison of MPRFs obtained in SPBs loaded with N/C-MPER_683_ or C-MPER-TMD_709_ peptides. Contact time: 0.5 s; Loading rate: 41 nN s^−1^. **d** Semi-logarithmic relation between MPRF and loading rate fitted with the Bell-Evans model. Means (±SD; *n* > 150) of the experimental MPRFs values measured with distinct loading-rates were fitted to linear regressions. Estimated *χ*_β_ and *k*_off_ values are displayed in the panels.

In our experiments, supported phospholipid bilayers (SPBs) loaded with reconstituted N/C-MPER_683_ or C-MPER-TMD_709_ peptides were deposited onto mica surfaces. To ensure that the SPB surfaces were homogeneous and defect-free, we first carried out their topographical characterization. AFM images displayed in Fig. 5a compare the topography of POPC SPBs devoid of peptide with those containing N/C-MPER_683_ or C-MPER-TMD_709_ reconstituted at 1:50 peptide-to-lipid mole ratio. In all instances, the heights of the flat membrane patches adhered to the mica substrate were consistent with the thickness of a single phospholipid bilayer (ca. 4 nm).

Diagrams depicted in the Supplementary Fig. 7a illustrate the force-extension experimental approach subsequently followed to evaluate antibody-epitope binding strength. The Fab 10E8 was covalently tethered via a crosslinker to AFM tips (Supplementary Fig. 7b). During the force-extension procedure, the AFM tip was cyclically brought into contact with the SPB surface, allowing the formation of an antibody-epitope complex. In such case, the retraction of the tip from the surface translated into an increasing force that applied continuously to the formed intermolecular complex until the Fab-epitope connection broke apart at a critical force (unbinding force).

Figure 5b displays experiments detecting the number of binding events as a function of the AFM tip-SPB contact time. Events were hardly discernable when SPBs devoid of peptide were probed. In contrast, not all but a significant fraction of the retraction curves (<20%) displayed unbinding events in the presence of peptides. Moreover, attachment appeared to occur at higher frequency in the case of the C-MPER-TMD_709_-containing SPBs (see also Supplementary Fig. 8).

Next, we determined Fab-peptide unbinding forces as a function of the loading rate^66,67^. We determined the most probable rupture force (MPRF) at a given retract velocity by fitting the frequency distribution of the rupture forces with a Gaussian model (i.e., the equivalent to a “force spectrum”). Figure 5c compares MPRFs obtained for SPBs loaded with N/C-MPER_683_ or C-MPER-TMD_709_ (top panels). The MPRF was clearly shifted to higher values in the C-MPER-TMD_709_ sample.

Finally, we turned to the Bell-Evans analysis to obtain the Fab-peptide dissociation *k*_off_ rates (Fig. 5c, bottom panels). To produce the Bell-Evans plots, MPRF determinations were repeated several times at each retract velocity and mean values plotted against the natural logarithm of the loading rate. The distance of the unbinding energetic barrier (*χ*_β_) could be obtained from the slope of the linear fit of the data, and the off-rate *k*_off_ deduced by linearly extrapolating the experimental data to zero external force (i.e., conditions of thermal energy alone). The values displayed in the plots confirmed comparable *χ*_β_ values for unbinding in both peptides, but 20-fold slower dissociation rate for the Fab-C-MPER-TMD_709_ complex, in comparison with the Fab-N/C-MPER_683_ complex.

### Effect of the reconstituted C-MPER helix on the stability of lipid bilayers

**Fig. 6:**
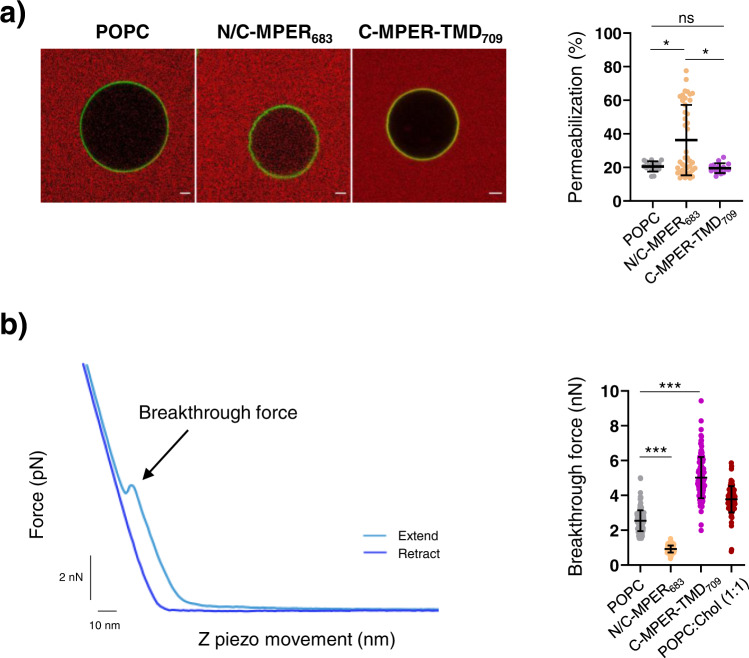
Effects of C-MPER helix reconstitution on the stability of lipid bilayers. **a** Stability measured in single-vesicle assays. Left: Micrographs of NBD-labeled single GUVs immersed in a solution containing the water-soluble KK114 fluorescent probe (red background). GUVs devoid of peptide are compared with those containing the N/C-MPER_683_ or C-MPER-TMD_709_ peptide reconstituted. The presence of red label inside the vesicles indicates effective permeabilization. Right: quantitation of permeabilization levels after 60 min incubation. Scale bars are 2 µm. Number of vesicles *n* ≥ 16. Center line, mean; whiskers SD (Mann–Whitney Test: ***p < 0.001; **p < 0.01, *p < 0.05, n.s. ≥ 0.05). **b** Effects on the breakthrough force of POPC SPBs. Left: Experimentally measured approaching and retraction force curves performed on a sample showing the jump in distance of ca. 4 nm with an applied force of 6 nN. Right: Mean breakthrough force values measured in the different samples. The N/C-MPER_683_-containing SPBs displayed significantly lower breakthrough forces than those containing C-MPER-TMD_709_. Number of measurements *n* ≥ 200. Center line, mean; whiskers SD (Mann–Whitney Test: ***p < 0.001; **p < 0.01, *p < 0.05, n.s. ≥ 0.05). The peptide-to-lipid mole ratio was 1:50 in the samples containing peptide. The POPC:Chol (1:1) sample was included as a control of membrane stiffening.

Alterations of the lipid bilayer material properties may distort the conformations and insertion modes of membrane-residing peptides^68^. In the context of peptide-based, anti-MPER immunogens formulated in lipid bilayers^17,39^, this might translate into a reduction of the fraction effective for antibody binding. To inquire whether the reconstituted C-MPER helices could perturb the stability of the lipid bilayer, we next determined peptide effects on the permeability barrier and mechanical stability of the containing membranes (Fig. 6).

Figure 6a (left) compares confocal micrographs of single GUVs lacking peptide with those loaded with N/C-MPER_683_ or C-MPER-TMD_709_. Untreated vesicles and vesicles-containing reconstituted C-MPER-TMD_709_ were viewed as empty spheres surrounded by the NBD-labeled lipid bilayer (depicted in green color), against a background containing the permeant KK114 fluorescent probe (rendered in red color). In contrast, a large fraction of the vesicles incorporating N/C-MPER_683_ showed different levels of red labeling in their internal compartments, indicative of solute permeation. This effect was quantitatively accounted for by measuring KK114 fluorescence intensities inside a population of the vesicles, which were consistent with the rupture of the permeability barrier (Fig. 6a, right).

Permeabilization of single vesicles correlated with effects of the reconstituted peptides on the nanomechanical stability of SPBs analyzed by FS-AFM^57^ (Fig. 6b). In this approach, we measured the breakthrough forces of the different SPBs to quantitatively determine the nanomechanical resistance to membrane rupture. By comparing the AFM tip indentations through the different bilayer systems at the same approach speed, we measured the discontinuity in the approach curve which corresponds to the breakthrough force; a parameter widely used to assign the mechanical stability of SPBs^57^. The obtained values indicated that reconstitution of N/C-MPER_683_ reduced the force required to break the POPC lipid bilayer, consistent with a reduction of its mechanical stability. Interestingly, inclusion of C-MPER-TMD_709_ increased the values of the forces at which rupture was observed. This increase was even larger than that observed in an SPB containing 50 mol % cholesterol, which was added as a stiffening-control sample to the analysis. Thus, in contrast to N/C-MPER_683_, C-MPER-TMD_709_ appeared to stiffen the lipid bilayer following the trend displayed by cholesterol. This different behavior could arise from effects on lipid packing modulated by the distinct topologies adopted by the peptides in the membrane, adsorbed onto the interface the former, and integrated as a membrane-spanning moiety the latter (see Discussion below).

## Discussion

HIV bnAbs against C-MPER consistently exert neutralization with extraordinary breath, therefore composing a gold standard for the class of antibodies that a preventive AIDS vaccine should aim at producing^4^. Accordingly, the most potent member of this class, 10E8, has recently become a focus for antibody optimization and immunotherapy^8,29,33,37,38,69,70^. BnAb 10E8 binds to its target C-MPER helical epitope in the environment of the membrane^27,29,33,34^. However, opposite to the view of a structurally stable epitope region engaged by the components of the immune system, insertion of MPER-based peptides into lipid bilayers appears to generate the type of perturbations required for membrane fusion^71–76^. This static (antigen) vs. dynamic (fusogen) behavior may reflect the existence of fluctuating structures and modes of MPER interactions with membranes, which are different in the pre-fusion states of the Env glycoprotein^11,16,25–27,30,34,77^, upon fusion activation^76^, or after the completion of the process^78,79^. Hence, discerning conditions that ensure the stable presentation of the 10E8 epitope to the immune system from the ones that promote bilayer perturbations is incumbent, not only to develop effective HIV-1 MPER vaccines, but also to design new molecular baits that will allow the identification of B-cells expressing potent 10E8-like bnAbs.

Our data comparing 10E8 recognition of C-MPER helices in the presence of DPC micelles confirmed higher affinity and specificity when the TMD section bound to the Fab in the crystal^29^ is present (Fig. 2). In combination, titration assays and resistance to thermal denaturation of Fab-peptide complexes suggest that the number of interactions sustaining the specific binding to the C-MPER helix increase after addition of TMD residues. One possibility is that the TMD scaffold ensures satisfaction of all possible specific interactions, including those established by residues at the tip of the HCDR3. In contrast, interrupting MPER-TMD sequence at residue K/R683 might result in less specific, non-polar contacts.

These observations are in agreement with SPR analyses comparing binding of detergent-solubilized MPER-TMD peptides to LN01 and 10E8 bnAbs^11^, which revealed higher affinity after incorporating the full TMD anchor, an effect that was more marked in the case of the former Ab. Interestingly, this higher affinity in presence of the full TMD might reflect a capacity of 10E8 and LN01 to engage with this moiety in the context of different fusion intermediates^78^. Also in line with these findings, inclusion of the N-terminal TMD section appears to increase the extent of 10E8 binding when an MPER-TMD continuous helix is fully exposed associated to the membrane interface in vesicles^24^.

Reconstitution into lipid bilayers of C-MPER helices with differing topologies further allowed evaluating the contribution of membrane-anchoring through the TMD to 10E8 binding. As a general rule, PC-based lipid bilayers containing reconstituted C-MPER-TMD helices appeared to expose 10E8 epitope efficiently, the degree of exposure being a function of the length of the membrane-spanning sequence and the bilayer thickness (Figs. 3, 4). In addition, spectroscopy data were consistent with the potential self-oligomerization of the TMD-containing peptides (Figs. 2, 3). However, we surmise that the adoption of a quaternary structure does not explain per se the observed increase in epitope accessibility in the reconstituted peptides for the following reasons: (i) one possible mechanism by which oligomers might become better ligands than monomers is binding avidity. Since we are working with Fabs containing single binding sites, and self-oligomerization of peptides is expected to reduce the density of epitope accessible in membranes (most likely reducing also apparent affinity), we discard this mechanism; (ii) other possibility is that the antibody recognizes a structure within the quaternary arrangement of MPER helices, essentially absent from monomers. This possibility is not supported by available structural data (X-ray diffraction and Cryo-EM) indicating that 10E8 engages with one face of a single MPER helix^8,29,33^. In addition, this mechanism would not explain the low, but still significant, level of binding to monomers observed in the case of MPER helices devoid of the TMD (Figs. 3–5).

Therefore, we favor the interpretation that potential oligomerization in membranes may help adopt the pole-like topology favorable for epitope recognition by the MPER-TMD peptides, but that the adoption of the correct topology is the crucial factor that actually conditions the process. In fact, the existence of an almost linear correlation between the hydrophobic mismatch and extents of Fab binding (Fig. 4c) allows inferring from simple geometrical considerations that the basis supporting Fab-epitope binding is the length of the C-MPER-TMD helix stretch exposed to solvent. For instance, similar levels of Fab-epitope binding are determined in C-MPER-TMD_700_/diC14:1PC and C-MPER-TMD_709_/diC22:1PC systems displaying equivalent hydrophobic mismatches (red asterisks in Fig. 4c).

To establish the differences in 10E8 epitope recognition depending on the helix topology, we additionally performed single-molecule measurements of Fab-peptide unbinding forces in SPBs (Fig. 5). The specificity of the unbinding events was supported by two observations: (i) the relatively low frequency at which the events were scored in the series of force-distance cycles; and (ii) the fact that virtually no events were detected when POPC SPBs devoid of peptide were probed. Furthermore, for similar membrane densities of the reconstituted peptides, contact time experiments suggested an epitope accessibility degree higher for the C-MPER-TMD_709_ peptide than for N/C-MPER_683_. In experiments carried out increasing the loading rate, the need to apply a greater force to break the Fab-peptide complex was also invariably detected in the case of C-MPER-TMD_709_. The data derived from the Bell-Evans diagram seem to indicate that indeed the rate of dissociation is slower for C-MPER-TMD_709_ peptide than for N/C-MPER_683_. However, the fact that the distance to the activation barrier is almost the same in both complexes, would be consistent with an interaction mechanism mediated at the molecular level by essentially identical binding-site structures.

Given the higher degree of accessibility observed when reconstituted in lipid bilayers, and previously reported evidences^24^, it seems unlikely that the rate of association (*k*_on_) to the peptide C-MPER-TMD_709_ diminishes greatly in comparison with that to N/C-MPER_683_. The values estimated for the interaction times needed for half maximal probability of binding (*t*_0.5_-s) support that assumption (Supplementary Fig. 8). Assuming that *t*_0.5_ values are roughly proportional to the *k*_on_ values, the ratio *t*_0.5_(N/C-MPER)/ *t*_0.5_(C-MPER-TMD) would amount to ≈1.4. This denotes *k*_on_ values in the same range, but slightly faster in the case of N/C-MPER. Taking into account the *k*_off_ ratio among peptides (≈22), the binding affinity ratio *K*_D_(N/C-MPER)/*K*_D_(C-MPER-TMD) would amount to ca. 15.

Thus, in comparison with its exposure as an interfacial helix, an antibody affinity at least 15-fold higher (*K*_D_ value 15-fold lower) may be expected for the 10E8 epitope when exposed as the N-terminal helix of the membrane-spanning TMD. When compared to the 10-fold increase in affinity observed in detergent micelles, it can be deduced that, besides imparting specificity to the interaction, the transmembrane topology of the TMD helix seems to play an additional role during the Fab-epitope binding process that evolves in the context of lipid bilayers.

One possible mechanism that could explain higher affinity for a pole-like 10E8 epitope is the existence of a membrane-accommodating surface in the variable domain of the Fab^29,33,35–37,44^. It has been postulated that driven by the specific recognition of the C-MPER epitope, this Fab surface would contact the membrane interface and establish favorable interactions therein, including semi-specific binding to phospholipid head groups^29,33^. These interactions could contribute to reduce the dissociation rates of Fab-epitope binding in the membrane context. Complementarily, Fab engagement with a C-MPER helix protruding from the membrane plane generates a belt of aromatic residues at the membrane interface that might promote further the stability of the Fab-epitope complex^29^.

Efficient exposure of the 10E8 epitope at membrane surfaces is expected to depend also on the overall stability of the lipid bilayer. Single-vesicle experiments revealed partial permeabilization of GUVs that contained N/C-MPER_683_ reconstituted. Consistently, FS-AFM measurements indicated that N/C-MPER_683_ incorporated at 1:50 peptide-to-lipid mole ratio reduced the breakthrough force of the bilayer. Being its location eminently interfacial, these effects could reflect the lipid packing defects generated by its insertion into a single monolayer. In sharp contrast, GUVs that contained C-MPER-TMD_709_ were essentially impermeable. Furthermore, the inclusion of the C-MPER-TMD_709_ helix exerted a clear effect of mechanical stabilization of the bilayer consistent with a higher degree of lipid packing.

Thus, measurements of bilayer stability indicated that N/C-MPER_683_ and C-MPER-TMD_709_ reconstituted adopting similar helical conformations but different topologies, affect the nanomechanical properties of the bilayer in opposite ways. The permeabilization and softening effects induced by N/C-MPER_683_ would be in line with the roles proposed for MPER-membrane interactions during the fusion process^14,15,21,74,79,80^. In contrast, the reconstituted C-MPER-TMD_709_ helix seems to represent better more stable pre-fusion states of the C-MPER epitope within the Env complex.

Most liposome-based vaccine approaches have assumed until now that synthetic peptides representing the MPER sequence (i.e., ending at position K/R683) can expose neutralizing epitopes in a fixed position when attached to the surface of vesicles^17,19,43,81^. However, even in cases where the overall architecture of vesicles remains uncompromised, the material properties of the lipid bilayer scaffold can change at the nanoscopic level by effect of peptide insertion into the interface, which in turn can modulate peptide insertion modes and conformations^68^.

In this study, we tested the possibility that Env TMD residues contribute crucially to C-MPER epitope recognition in membranes. Collectively, our data support that: (i) TMD residues confer higher affinity and specificity to 10E8 binding; (ii) in comparison with an interface-adsorbed helix, the 10E8 epitope is exposed more efficiently for engagement with antibody when membrane-anchored through TMD residues; and (iii) lipid bilayers that contain the C-MPER epitope anchored through the TMD are mechanically more stable than the ones containing an interfacial MPER helix. Thus, we conclude that to produce 10E8-like Abs through vaccination, inclusion of Env TMD residues would be beneficial in all types of formulations, including micellar systems, where TMD residues are not acting as an anchor to the lipid bilayer. We infer that the double effect of increasing specificity and providing a defined topology, further observed when the full TMD moiety anchors the C-MPER epitope to the lipid bilayer, might be crucial to select for B-cells producing neutralizing antibodies that can accommodate the lipid bilayer optimally.

## Methods

### Reagents

The peptide sequences derived from the gp41 MPER-TMD region displayed in Figure 1b were produced as C-terminal carboxamides by solid-phase synthesis using Fmoc chemistry and purified by HPLC. PC-based synthetic phospholipids and dodecylphosphocholine (DPC) were purchased from Avanti Polar Lipids (Birmingham, AL, USA). N-(7-Nitrobenz-2-Oxa-1,3-Diazol-4-yl)-1,2-Dihexadecanoyl-sn-Glycero-3-Phosphoethanolamine (NBD-PE) was from Thermo Fisher Scientific (Waltham, Massachusetts, USA). Abberior STAR RED (KK114) was obtained from Abberior (Göttingen, Germany). 1,1,1,3,3,3-hexafluoro-2-propanol (HFIP) was obtained from Sigma-Aldrich (St. Louis, Missouri, USA).

### Production of Fab 10E8

Fab 10E8 antibody sequence was cloned in the plasmid pColaDuet and expressed in *Escherichia coli* T7-shuffle strain. Recombinant expression was induced at 18 °C overnight with 0.4 mM isopropyl-D-thiogalactopyranoside when the culture reached an optical density of 0.8. Cells were harvested and centrifuged at 8000 *× g*, after which they were resuspended in a buffer containing 50 mM HEPES (pH 7.5), 500 mM NaCl, 35 mM imidazole, DNase (Sigma-Aldrich, St. Louis, MO) and an EDTA-free protease inhibitor mixture (Roche, Madrid, Spain). Cell lysis was performed using an Avestin Emulsiflex C5 homogenizer. Cell debris was removed by centrifugation, and the supernatant loaded onto a nickel-nitrilotriacetic acid (Ni-NTA) affinity column (GE Healthcare, Chicago, Illinois, USA). Elution was performed with 500 mM imidazole, and the fractions containing the His-tagged proteins were pooled, concentrated and dialyzed against 50 mM sodium phosphate (pH 8.0), 300 mM NaCl, 1 mM DTT, and 0.3 mM EDTA in the presence of purified protease Tobacco etch virus^82^. Fabs were separated from the cleaved peptides containing the His_6x_ tag by an additional step in a Ni-nitrilotriacetic column. The flow-through fraction containing the antibody was dialyzed overnight at 4 °C against sodium acetate (pH 5.6) supplemented with 10% glycerol and subsequently loaded onto a MonoS ion exchange chromatography (IEC) column (GE Healthcare, Chicago, Illinois, USA). Elution was carried out with a gradient of potassium chloride and the fractions containing the purified Fab concentrated and dialyzed against a buffer containing 10 mM sodium phosphate (pH 7.5), 150 mM NaCl, and 10% glycerol. For confocal microscopy experiments, position C216_HC_ of the Fab was modified in vitro with a sulfhydryl-specific iodacetamide derivative of the KK114 probe.

### Circular dichroism

Circular dichroism (CD) measurements were carried out on a thermally-controlled Jasco J-810 circular dichroism spectropolarimeter calibrated routinely with (1 S)-(+)−10-camphorsulfonic acid, ammonium salt. Peptides were dissolved in an aqueous buffer (2 mM Hepes, pH, 7.4) at 0.03 mM concentration with increasing concentrations of DPC. Spectra were measured in a 1 mm path-length quartz cell equilibrated at 25 °C. Data were taken with a 1 nm band-width, 100 nm/min speed, and the results of 20 scans per sample were averaged.

### Thermodynamic assays

Isothermal titration calorimetry (ITC) experiments were performed with a VP-ITC microcalorimeter (MicroCal, Northampton, MA) at 25 °C. To avoid “buffer mismatch”, prior to the experiment, proteins were dialyzed overnight at 4 °C against 10 mM sodium phosphate (pH 7.5), 150 mM NaCl. To assure the stability of the antibody, the dialysis buffer was further supplemented with 10% (v/v) glycerol. Samples containing protein and peptide solubilized in dialysis buffer were supplemented with 5 mM DPC and degassed immediately before each measurement. Fab10E8 (3 μM) was titrated with peptide (40 μM). The volume of each injection was 10 μL. Peptide dilution heat was subtracted for data analysis. The binding isotherms were fitted to a one-site binding model using the program ORIGIN 7.0 (MicroCal, Northampton, MA). The fitting procedure yields the stoichiometry (*n*), the binding constant (*K*_D_) and the enthalpy (*ΔH*) of the binding reaction. For differential scanning calorimetry (DSC) determinations, heat capacity was measured using a VP-DSC scanning microcalorimeter (MicroCal, Northampton, MA). Temperature scans were performed in a 10 mM sodium phosphate (pH 7.5), 150 mM NaCl, and 10% glycerol buffer supplemented with 5 mM DPC. Samples of 10E8 (10 μM) with and without peptide (15 μM) were heated from 30 to 90 °C at a rate of 1 °C min^−1^. The ORIGIN software package (MicroCal) was used for data collection and analysis. The buffer baseline was subtracted from the sample raw data, normalized by protein concentration, and fitted with a two-state thermal transition model to obtain thermodynamic parameters

### Reconstitution of C-MPER into lipid bilayers

To prepare lipid bilayers containing reconstituted peptide, lipids and MPER-TMD-derived peptides were mixed in organic solvent prior to the production of the liposomes. Briefly, POPC dissolved in chloroform:methanol 1:2 (vol:vol) was mixed with peptide dissolved in HFIP at the desired peptide-to-lipid molar ratio. The mixture was dried under a N_2_ stream and traces of organic solvents were removed by 1 h vacuum pumping. Subsequently, the dried lipid films were subjected to 2 h of gentle hydration with H_2_O using a N_2_ gas bubbler to facilitate further dispersion of the dried lipid-peptide film in PBS aqueous buffer. Next, the multilamellar vesicles were bath sonicated (1 h, 55 °C) and subjected to 15 freeze and thaw cycles to obtain unilamellar vesicles.

### Infrared spectroscopy

Infrared spectra were recorded in a Bruker Tensor 27 spectrometer equipped with a mercury-cadmium-telluride detector using a Peltier-based temperature controller (TempCon, BioTools Inc., Wauconda, IL) with calcium fluoride cells (BioCell, BioTools Inc., Wauconda, IL). Lipid vesicles with the peptides reconstituted were lyophilized and subsequently prepared at 3 mg/mL in D_2_O buffer (PBS). A 25 μL sample aliquot was deposited on a cell that was sealed with a second cell. Reference windows without peptide were prepared similarly. Typically, 1000 scans were collected for each background and sample, and the spectra were obtained with a nominal resolution of 2 cm^−1^. Data treatment and band decomposition of the original amide I have been described elsewhere^83^.

### Binding and permeability measurements in single vesicles

For binding experiments, NBD-labeled GUVs were produced following the electro-formation method as previously described^47^. In brief, a total of 2 mM of lipid was dissolved in 200 μL CHCl3:CH3OH with the fluorescent probe NBD-PE (0.5%). When required, peptide dissolved in 10% (v/v) HFIP was included in the organic phase at 1:250 peptide-to-lipid molar ratio. The GUVs were added to a bovine serum albumin (BSA)-blocked microscope chamber that already included 250 nM of 10E8-based Fabs conjugated with the KK114 probe at residue C216_HC_, and were incubated for 15 min prior to imaging. For GUV permeabilization assays, GUVs were added to a BSA-blocked microscope chamber that already included the unconjugated and soluble KK114 dye, and were incubated for 15 min prior to imaging. Confocal microscopy images were acquired on an inverted confocal fluorescence microscope (Nikon Eclipse TE-2000, Nikon, Nikon Instruments, Tokyo, Japan). NBD-stained GUVs and Fabs conjugated with the KK114 probe were excited at 476 nm and 637 nm, respectively. Under these measuring conditions, fluorescence emission levels were negligible in samples incubated with unlabeled antibody. The band pass filters used were 515/30 and Long Pass 650 nm. The objective used was a 63X oleo immersion with a numerical aperture (NA) of 1.2.

### Atomic force microscopy

SPBs were produced by the vesicle adsorption method and their topography analyzed using a Nanowizard III AFM (JPK Instruments) under contact mode. Briefly, approximately 30 µl of the vesicle suspensions described above were deposited onto freshy cleaved mica disks (area 15 mm^2^) preincubated with 30 µl of 150 mM KCl, 10 mM Tris, pH = 7.4 (measurement buffer) and incubated for 30 min at room temperature, leading to the formation of SPBs. The samples were carefully rinsed with measurement buffer before the AFM experiment and always kept under aqueous environment. BL-AC40-C2 Si_3_N_4_ cantilevers (Olympus, Japan) with a nominal spring constant of 0.09 N/m were used for bilayer imaging with 0.5 nN of force applied.

Prior to imaging, cantilevers were individually calibrated in a lipid free mica substrate in buffer, using the thermal noise method, after having correctly measured the piezo sensitivity (V/m).

For single molecule force spectroscopy, MLCT-BIO probes (Bruker, Santa Barbara, CA USA) were functionalized with Fab 10E8 using well established protocols^84^. To that end, cantilevers were first rinsed with acetone and water, exposed to UV-ozone and coated with 3-Aminopropyldimethylethoxysilane (APDMES). They were then incubated with glutaraldehyde and finally with Fab. Finally, to passivate possible unreacted aldehyde moieties, cantilevers were incubated in 0.1% Bovine Serum Albumin (BSA) for 1 h at room temperature and rinsed in measurement buffer prior to use. Force maps were recorded using a maximum applied force of 0.2 nN, a varying contact time of 0.5, 1, 2 and 5 s and varying retraction speeds of 0.1 µm/s, 2 µm/s and 10 µm/s. Data were analyzed with the data processing software from JPK Instruments (Berlin, Germany).

To determine the MPRF at a given loading rate the frequency histograms of the measured forces were fitted to a Gaussian distribution (Eq. 1) and its maximum calculated.

1 $$y={Amplitude} \cdot {{\exp }}\left(-0.5 \cdot {\left(\frac{x-{Mean}}{{SD}}\right)}^{2}\right)$$

MPRF is a function of the loading rate as defined by (Eq. 2):

2 $${MPRF}=\frac{{k}_{\beta } \cdot T}{{\chi }_{\beta }}{ln}\frac{{r}_{f} \cdot {\chi }_{\beta }}{{k}_{{off}} \cdot {k}_{\beta } \cdot T}$$

where *k*_β_ is Boltzmann constant, *T* is the temperature, *k*_off_ is the natural dissociation rate at zero force and r_f_ is the loading rate.

A linear fit of the MPRF data vs. the natural logarithm of the loading rate using (Eq. 3) reveals the distance of the unbinding energetic barrier from the equilibrium position (*χ*_β_) and the natural dissociation rate at zero force (*k*_off_).

3 $$y={y}_{0}+{ax}$$

These parameters can be actually obtained from the slope of the linear fit of the data, and by linearly extrapolating the experimental data to zero external force, respectively.

Force spectroscopy data for SPB breakthrough measurements were collected using SNL10 Si_3_N_4_ cantilever (Bruker, Santa Barbara, CA, USA) with a nominal spring constant of 0.35 N/m, at a speed of 1 µm/s and applying 10 nN of maximum force. Discontinuities in the force versus separation approach curve were determined for each of the indentation curves as reproducible jumps within the extended traces using JPK data processing software.

### Statistics and reproducibility

Statistical analysis was performed using GraphPad Prism 8.3. The statistical significance of all datasets was calculated using the non-parametric unpaired two-tailed Mann–Whitney test. Data are represented as mean ± S.D. Sample size is specified in figure legends. For all figures: ns ≥ 0.05; *p < 0.05; **p < 0.01; ***p < 0.001.

### Reporting summary

Further information on research design is available in the Nature Research Reporting Summary linked to this article.

## Supplementary information

Supplemental Material

Description of Additional Supplementary Data

Supplementary Data 1

Reporting summary

## Data Availability

The datasets generated during and/or analyzed during the current study are provided as Supplementary Data 1. ^1^H and ^13^C chemical shifts and the structures calculated for C-MPER-TMD_690_ in solutions containing DPC or HFIP have been deposited in the Biological Magnetic Resonance Data Bank (BMRB accession ID 51528 and 51531, respectively) and the Worldwide Protein Data Bank (wwPDB accession codes 8B6X and 8B6Y, respectively).
