# Supplementary material for: Molecular recognition of a membrane-anchored HIV-1 pan-neutralizing epitope
Source: Commun Biol. 2022 Nov 18;5:1265. doi: 10.1038/s42003-022-04219-6 (PMC9674588; doi:10.1038/s42003-022-04219-6)
Supplement: Supplementary file 1 — Supplemental Material [file 42003_2022_4219_MOESM1_ESM.pdf]

Supplementary Information

Molecular recognition of a membrane-anchored HIV-1 pan-neutralizing epitope

Johana Torralba[1,2], Igor de la Arada[1], Angélica Partida-Hanon[3], Edurne Rujas[1,2,], Madalen Arribas[1], Sara Insausti[1,2], Claire Valotteau[4], Javier Valle[5], David Andreu[5], José M.M. Caaveiro[6], María Angeles Jiménez[3], Beatriz Apellániz[7*], Lorena Redondo-Morata[8*] and José L. Nieva[1,2*]

[1]Instituto Biofisika (CSIC-UPV/EHU), University of the Basque Country (UPV/EHU), PO Box 644, 48080 Bilbao, Spain.

[2]Department of Biochemistry and Molecular Biology, University of the Basque Country (UPV/EHU), PO Box 644, 48080 Bilbao, Spain.

[3]Institute of Physical Chemistry "Rocasolano" (IQFR-CSIC), Serrano 119, E-28006 Madrid, Spain

[4]Laboratoire Adhesion et Inflammation, UMR INSERM 1067, UMR CNRS 7333, Aix-Marseille Université, Turing Centre for Living systems, Marseille, France

[5]Laboratory of Proteomics and Protein Chemistry, Department of Medicine and Life Sciences, Pompeu Fabra University, Barcelona Biomedical Research Park, Dr. Aiguader 88, 08003 Barcelona, Spain

[6]Laboratory of Global Healthcare, School of Pharmaceutical Sciences, Kyushu University, Fukuoka 819-0395, Japan

[7]Department of Physiology, Faculty of Pharmacy, University of the Basque Country (UPV/EHU), Paseo de la Universidad, 7, 01006 Vitoria-Gasteiz, Spain.

[8]Université de Lille, CNRS, Inserm, CHU Lille, Institut Pasteur de Lille, U1019-UMR9017/CIIL-Centre d'Infection et d'Immunité de Lille, F-59000 Lille, France

Supplementary Methods

Recording of NMR spectra

To prepare the NMR samples approximately 1-2 mg of the lyophilized peptide C-MPER-TMD_{690} were dissolved in 0.5 mL of 2 mM HEPES buffer at pH 7.0 in H_2O/D_2O (9:1 ratio by volume) or in D_2O and containing either 25 % (v/v) 1,1,1,3,3,3-hexafluoro-2-propanol (HFIP-D2, 98%; Cambridge Isotopes Lab) or 20 mM deuterated dodecylphosphocholine (DPC-D38, 98%; Cambridge Isotopes Lab). Peptide concentration was ~0.9-1.8 mM. pH was measured with a glass micro electrode, not corrected for isotope effects and, when necessary, adjusted by adding minimal amounts of NaOD or DCl. All samples contained sodium 2,2-dimethyl-2-silapentane-5-sulfonate (DSS) as internal reference for ^{1}H chemical shifts. Temperature of the NMR probe was calibrated using a methanol sample. ^{13}C δ-values were indirectly referenced by using the IUPAC-IUB recommended $^{1}H/^{13}C$ chemical shift ratio (0.25144953; [1]).

NMR spectra were recorded on Bruker Avance-600 spectrometer, equipped with a cryoprobe. As in the case of previously studied MPER peptides [2], 2D phase-sensitive two-dimensional correlated spectroscopy (COSY), total correlated spectroscopy (TOCSY), and nuclear Overhauser enhancement spectroscopy (NOESY) spectra were acquired by standard techniques using presaturation of the water signal and the time-proportional phase incrementation mode. NOESY mixing times were 150 ms, and TOCSY spectra were recorded using 60 ms DIPSI2 with z filter spin-lock sequence. ^{1}H-^{13}C heteronuclear single quantum coherence (HSQC) spectra were acquired at ^{13}C natural abundance using standard pulse sequences [2]. Acquisition data matrices were defined by 2048 x 512 points in t_2 and t_1, respectively. Data were processed using the standard TOPSPIN program (Bruker Biospin, Karlruhe, Germany). The 2D data matrix was multiplied by either a square-sine-bell or a sine-bell window function with the corresponding shift optimised for every spectrum and zero-filled to a 2K x 1K complex matrix prior to Fourier transformation. Baseline correction was applied in both dimensions.

NMR spectra assignment

^{1}H chemical shifts of C-MPER-TMD_{690} in each solvent conditions were assigned using the standard sequential assignment strategy [3] with the aid of the SPARKY software (T. D. Goddard and D. G. Kneller, SPARKY 3, University of California, San Francisco). ^{13}C chemical shifts were determined by analysis of ^{1}H-^{13}C-HSQC spectra. ^{1}H and ^{13}C chemical shifts were deposited at the BioMagResBank under accession codes: 51528 and 51531.

Structure calculation

Structures for peptide C-MPER-TMD_{690} in DPC micelles and in 25 % HFIP were calculated from distance and dihedral angle constraints derived from NMR parameters using the standard iterative procedure for automatic NOE assignment of the program CYANA 2.1 [4]. Distance constraints were obtained from the cross-peaks present in 150 ms 2D [^{1}H-^{1}H]-NOESY spectra, which were integrated using the standard SPARKY integration sub-routine (T. D. Goddard and D. G. Kneller, SPARKY 3, University of California, San Francisco), and the dihedral angle restraints for ϕ and ψ angles were derived from $^{1}H_{\alpha}$, $^{13}C_{\alpha}$ and $^{13}C_{\beta}$ chemical shifts using the TALOS-N webserver (http://spin.niddk.nih.gov/bax/nmrserver/talosn/)[5]. Since no ambiguous constraints were found upon examination of the list of distance constraints resulting from the last automatic cycle, the 20

lowest target function structures of this cycle were selected as the final structure ensembles. These ensembles were deposited in the Worldwide Protein Data Bank (wwPDB accession codes for C-MPER-TMD$_{690}$ in DPC micelles and in the presence of HFIP are 8B6X and 8B6Y, respectively). The structural statistics data for these structures are provided in Table 1 of the main text. The structures were visualized and examined using the programs MOLMOL [6].

Supplementary Figures

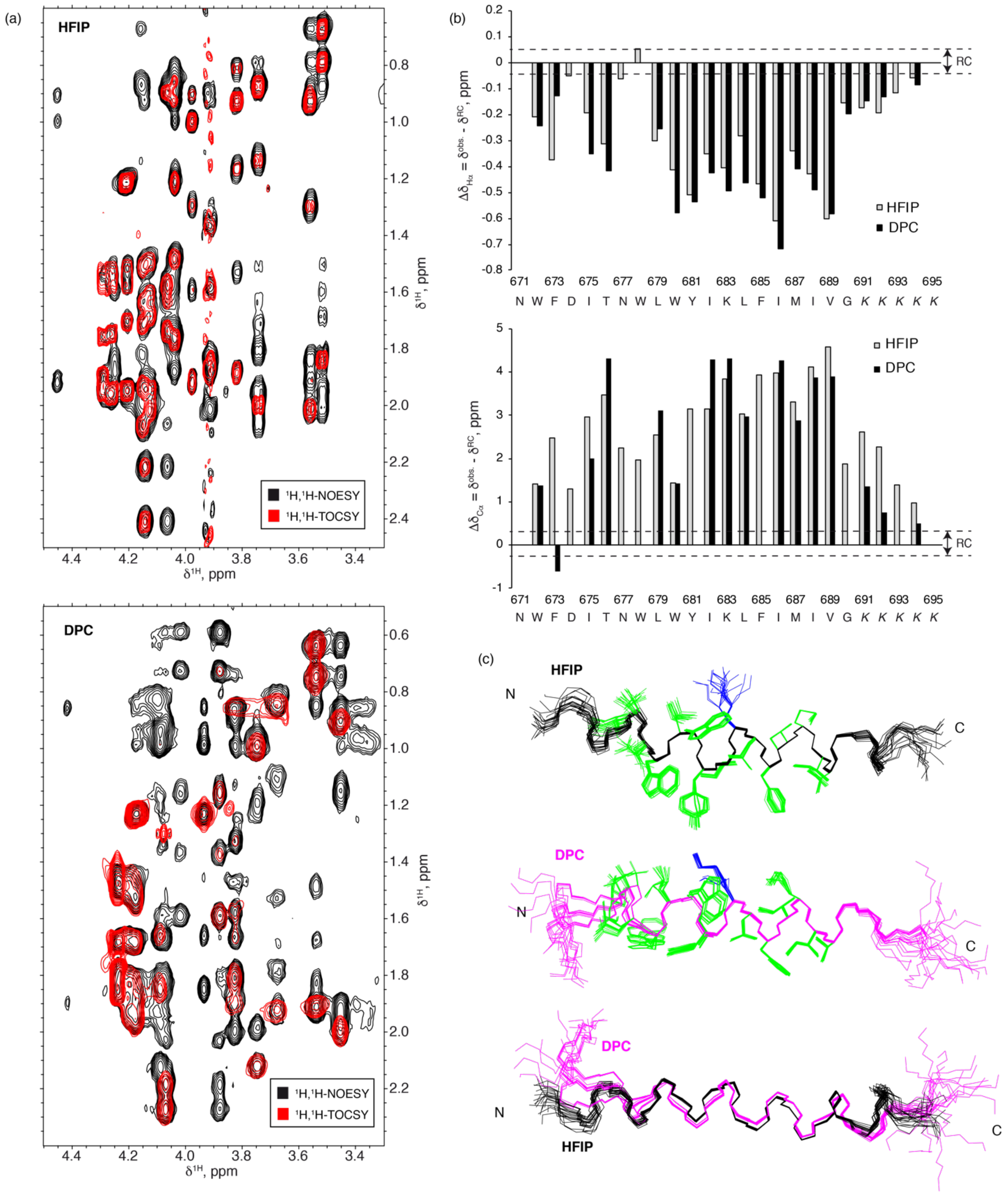

Supplementary Figure 1: NMR parameters for C-MPER-TMD$_{690}$ in non-polar media. (a) Selected regions of the 2D ^{1}H,^{1}H-NOESY spectra (black contours) recorded for C-MPER-TMD$_{690}$, onto which the 2D ^{1}H,^{1}H-TOCSY spectra (red contours) are overlaid. Spectra were obtained in 25 % HFIP (left) and in 20 mM DPC (right) in D_2O at pH 7.0 and 35ºC. The NOESY cross-peaks coincident with TOCSY cross-peaks correspond to intra-residue NOEs, and all other NOESY cross-peaks correspond to non-sequential NOEs, such as dαβ(i,i+3), characteristic of helical structures **(b)** Bar graphs showing $\Delta\delta_{H\alpha}$ ($\Delta\delta_{H\alpha} = \delta_{H\alpha}^{observed} - \delta_{H\alpha}^{RC}$, ppm) and $\Delta\delta_{C\alpha}$ ($\Delta\delta_{C\alpha} = \delta_{C\alpha}^{observed} - \delta_{C\alpha}^{RC}$, ppm) values (**top** and **bottom** panels, respectively) as a function of sequence in 25 % HFIP (black bars) or 20 mM DPC (white bars) at pH 7.0 and 35ºC. Negative $\Delta\delta_{H\alpha}$ values of large magnitude ($|\Delta\delta_{H\alpha}| > 0.05$ ppm) and large positive $\Delta\delta_{C\alpha}$ values ($|\Delta\delta_{C\alpha}| > 0.5$ ppm) are indicative of helical conformations. Dashed lines indicate the random coil (RC) ranges. Random coil values for H_α protons and C_α carbons were taken from Wishart et al.[7]. The N- and C-terminal residues are excluded because of charged end effects. (c) 3D structural ensembles calculated on the basis of NMR experimental restraints in 25% HFIP (top) and in DPC micelles (middle). Bottom panel shows the superposition of backbone atoms for the structural ensembles in HFIP (black) and in DPC (magenta). In top and middle panels, side chains for residues 675-690 are displayed in green, and that of K683 in blue.

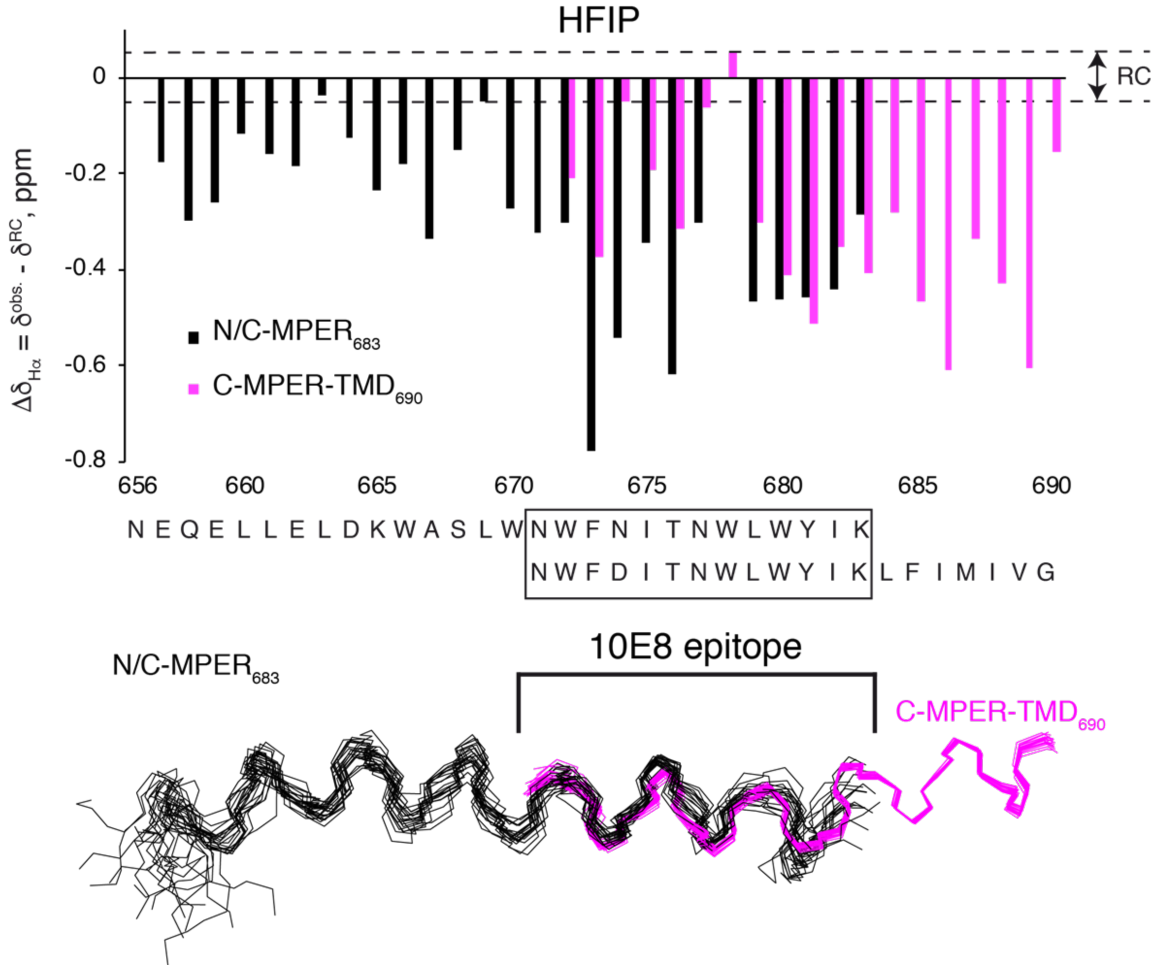

Supplementary Figure 2: Comparison of NMR structures of N/C-MPER$_{683}$[2] and C-MPER-TMD$_{690}$ in presence of 25 % HFIP. Top: Hα conformational shifts ($\Delta\delta_{H\alpha}$, ppm) observed for both peptides in 25 % HFIP, pH 7.0. Dashed lines indicate the random coil (RC) range. Bottom: superposition of the 3D structural models supporting the adoption of similar conformations by the 10E8 epitope sequence in both peptides.

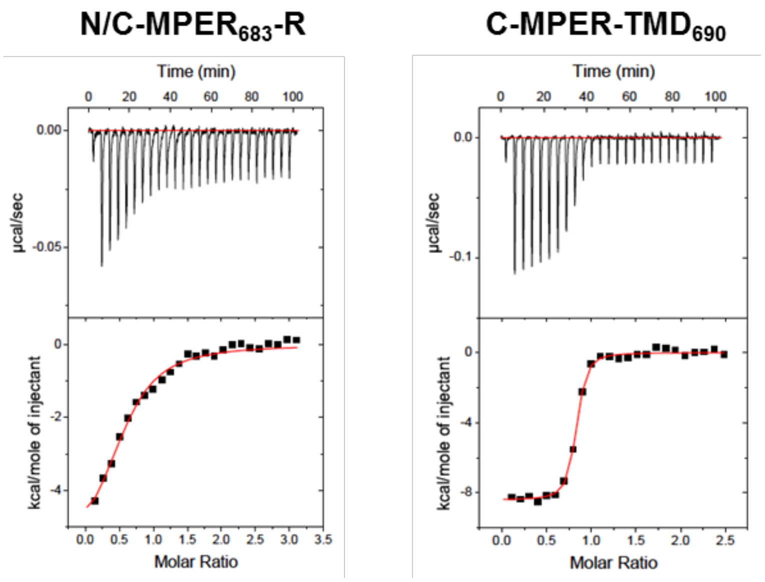

Supplementary Figure 3: Comparison of N/C-MPER683-R and C-MPER-TMD690 binding to Fab 10E8 by ITC. The upper panels indicate the heat released upon consecutive injections of 10 µL of peptide solution (40 µM) into Fab solution (3 µM) in the calorimeter cell in the presence of 5 mM DPC; the lower panels depict the integrated heats (symbols) and non-linear least-squares fit (line) of the data to a one-site binding model using ORIGIN 7.0. The data corresponding to C-MPER-TMD690 were adapted from a previous study by Rujas *et al.*[8].

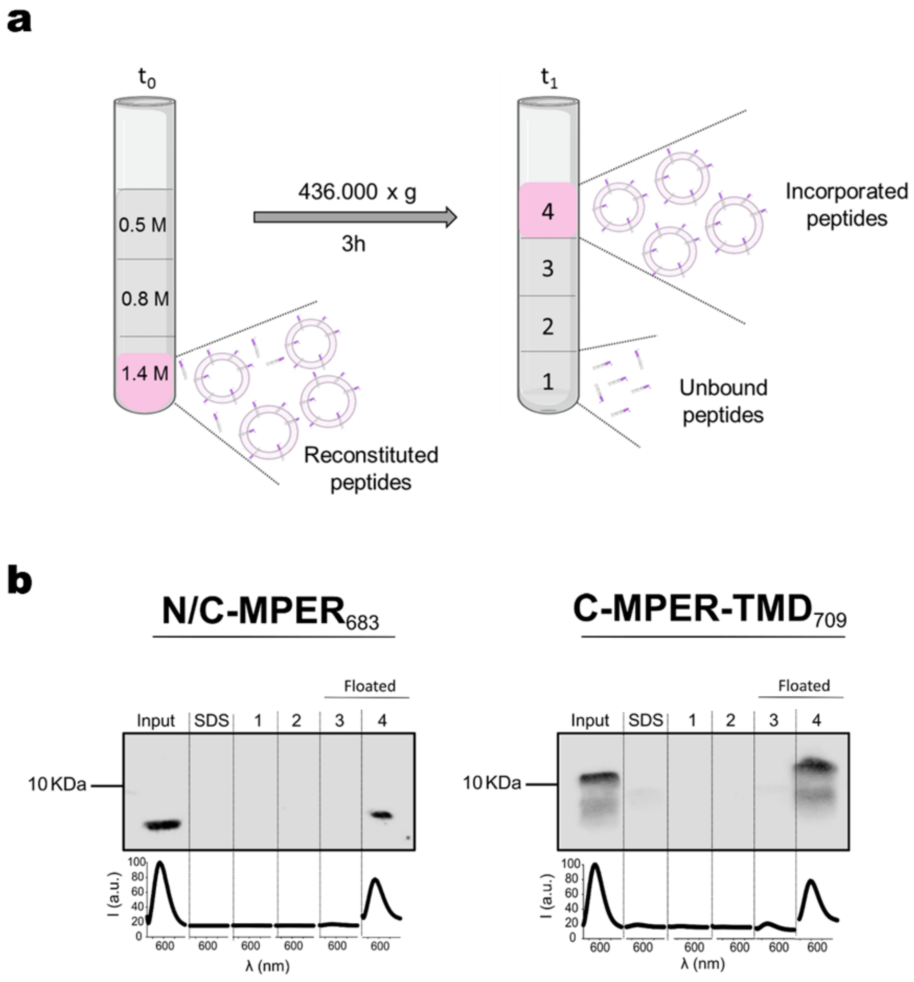

Supplementary Figure 4: Incorporation of reconstituted peptides into membranes. (a) Diagram illustrating the Liposome flotation method. Liposome-peptide complexes were subjected to ultracentrifugation in a sucrose gradient after the reconstitution process. **(b)** The presence of the peptides in the floated and non-floated fractions of the gradient and in the original sample (input) as revealed by Western Blot analysis after Tris-Tricine SDS-PAGE separation. Line plots below the panels display the fluorescence emission of the Rhodamine probe, revealing the presence of liposomes. The peptide-to-lipid ratio was 1:50 (mol:mol).

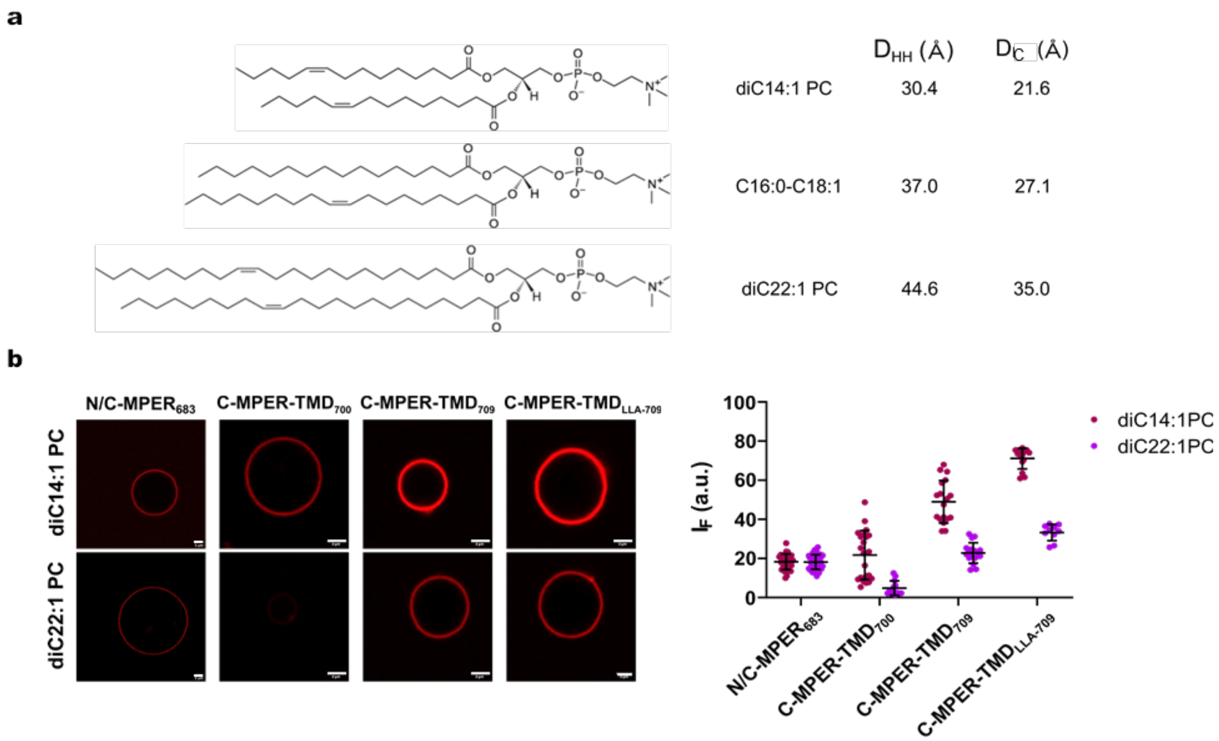

Supplementary Figure 5: Binding of Fab 10E8 to C-MPER helices reconstituted in 'thin' and 'thick' lipid bilayers. (a) Comparison of diC14:1PC and diC22:1PC with POPC (C16:0-C18:1PC). **(b)** Left: Confocal images at the equatorial plane of peptide-containing diC14:1PC and diC22:1PC GUVs, comparing binding of the fluorescently labeled KK114-10E8 Fab (rendered in red). All micrographs were rendered with equal contrast and brightness to best appreciate the difference in emission intensity. Right: KK114 fluorescence intensity quantification at the equatorial plane of individual GUVs in each sample. Scale bars are 2 µm. Number of vesicles $n \geq 30$. Center line, mean; whiskers SD.

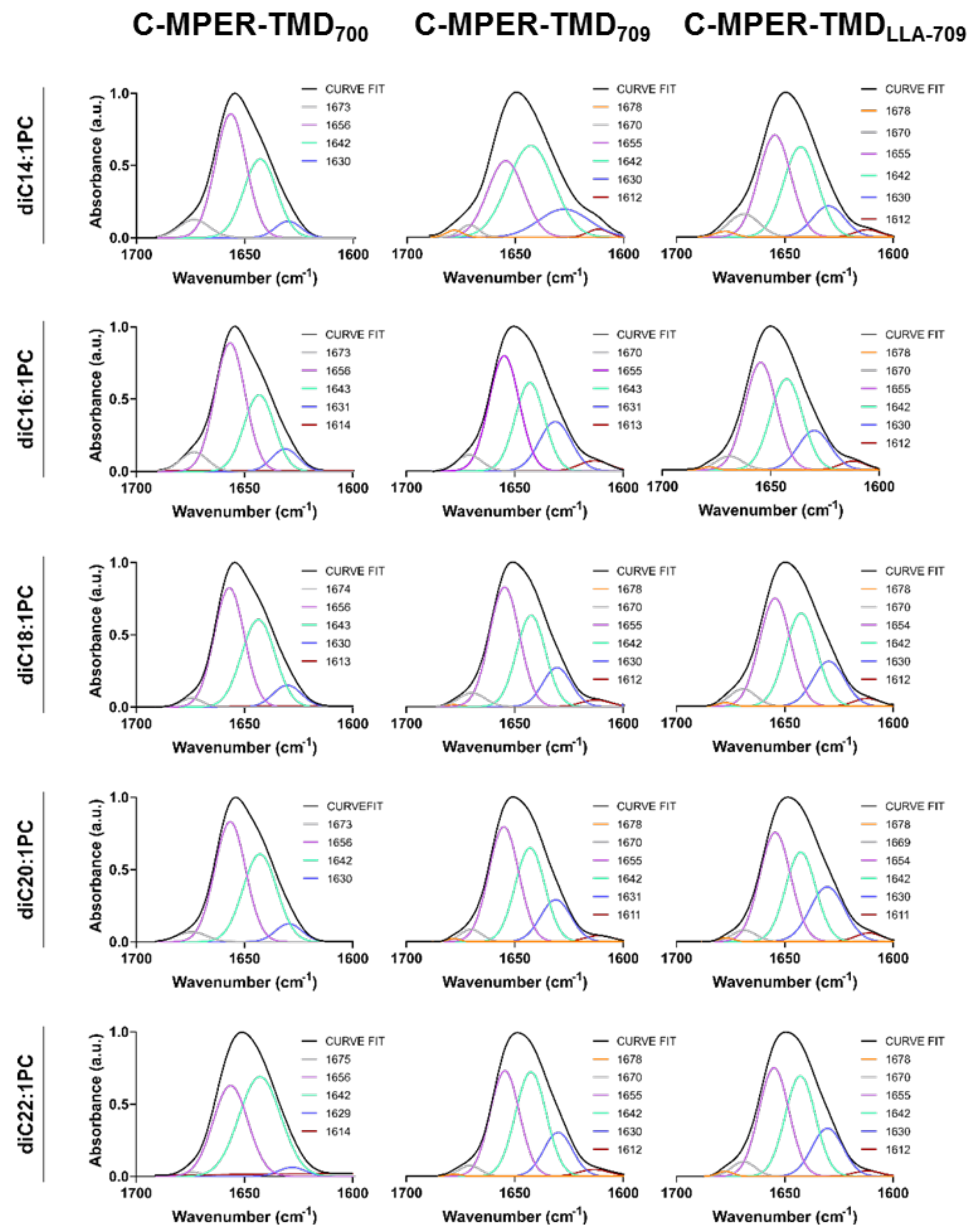

Supplementary Figure 6: IR spectra components (amide-I region) of C-MPER-TMD helices reconstituted in lipid bilayers of different thicknesses.

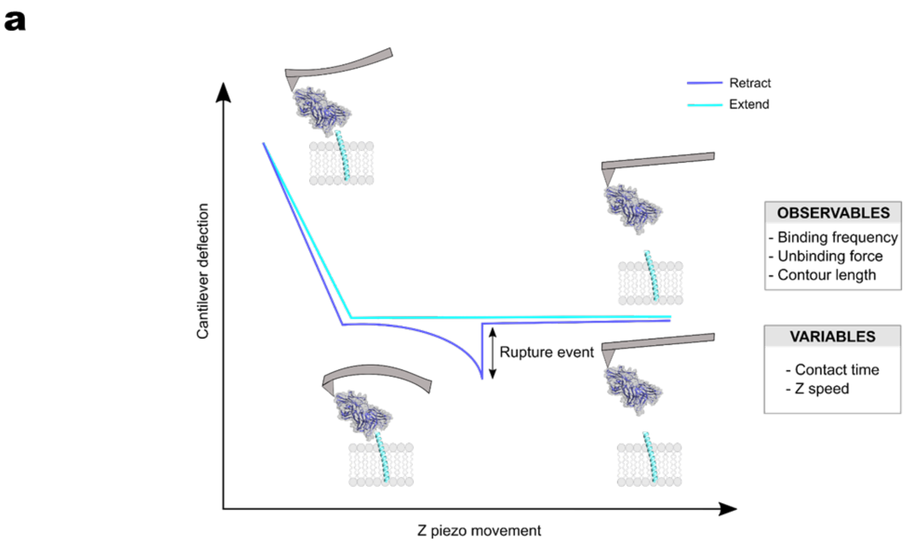

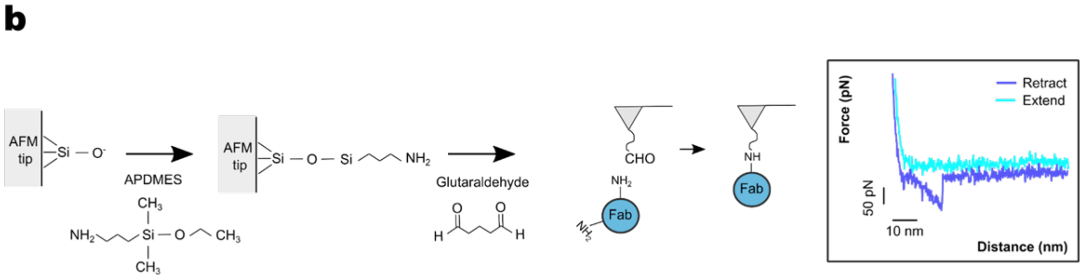

Supplementary Figure 7: Force-extension approach followed to detect single-Fab binding to MPER in SPBs. (a) Diagram depicting theoretical force-extension curves using AFM tips with Fab 10E8 covalently tethered via a crosslinker, and the parameters that can be inferred as a function of the measuring conditions. **(b)** Linking of Fab through free amines. Panel on the right displays a force-distance cycle revealing an unbinding event in POPC SPBs loaded with the C-MPER-TMD$_{709}$ peptide

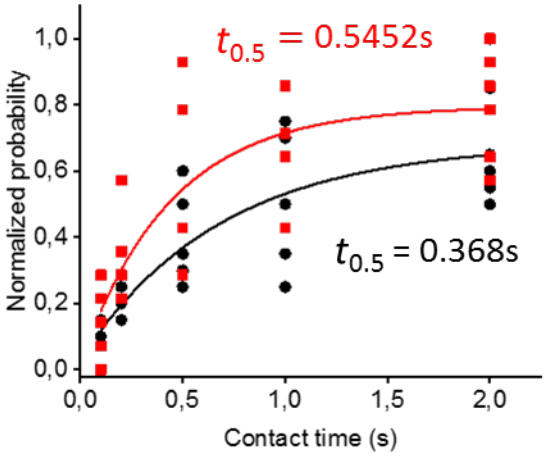

Supplementary Figure 8: Normalized probability vs contact time (s) for POPC:N/C-MPER$_{683}$ (black dots) and POPC:C-MPER-TMD$_{709}$ (red squares). Lines corresponding to exponential fittings to the data were used to estimate the times needed for half maximal probability of binding ($t_{0.5}$ values indicated in the panel).

.

Supplementary References:

1 Markley, J. L. *et al.* Recommendations for the presentation of NMR structures of proteins and nucleic acids. *J Mol Biol* **280**, 933-952, doi:10.1006/jmbi.1998.1852 (1998).

2 Serrano, S. *et al.* Structure and immunogenicity of a peptide vaccine, including the complete HIV-1 gp41 2F5 epitope: implications for antibody recognition mechanism and immunogen design. *J Biol Chem* **289**, 6565-6580, doi:10.1074/jbc.M113.527747 (2014).

3 Markley, J. L. *et al. Pure Appl. Chem.* **70**, 117-142 (1998).

4 Guntert, P., Mumenthaler, C. & Wuthrich, K. Torsion angle dynamics for NMR structure calculation with the new program DYANA. *J Mol Biol* **273**, 283-298 (1997).

5 Shen, Y. & Bax, A. Protein backbone and sidechain torsion angles predicted from NMR chemical shifts using artificial neural networks. *J Biomol NMR* **56**, 227-241, doi:10.1007/s10858-013-9741-y (2013).

6 Koradi, R., Billeter, M. & Wuthrich, K. MOLMOL: a program for display and analysis of macromolecular structures. *J Mol Graph* **14**, 51-55, 29-32 (1996).

7 Wishart, D. S., Bigam, C. G., Holm, A., Hodges, R. S. & Sykes, B. D. 1H, 13C and 15N random coil NMR chemical shifts of the common amino acids. I. Investigations of nearest-neighbor effects. *J Biomol NMR* **5**, 67-81 (1995).

8 Rujas, E. *et al.* Structural basis for broad neutralization of HIV-1 through the molecular recognition of 10E8 helical epitope at the membrane interface. *Sci Rep* **6**, 38177, doi:10.1038/srep38177 (2016).
